# The Use of Multiple Correspondence Analysis to Explore Associations between Categories of Qualitative Variables in Healthy Ageing

**DOI:** 10.1155/2013/302163

**Published:** 2013-10-09

**Authors:** Patrício Soares Costa, Nadine Correia Santos, Pedro Cunha, Jorge Cotter, Nuno Sousa

**Affiliations:** ^1^Life and Health Sciences Research Institute (ICVS), School of Health Sciences, University of Minho, 4710-057 Braga, Portugal; ^2^ICVS/3B's, PT Government Associate Laboratory, Guimarães, 4710-057 Braga, Portugal; ^3^Centro Hospital Alto Ave, EPE, 4810-055 Guimarães, Portugal

## Abstract

The main focus of this study was to illustrate the applicability of multiple correspondence analysis (MCA) in detecting and representing underlying structures in large datasets used to investigate cognitive ageing. Principal component analysis (PCA) was used to obtain main cognitive dimensions, and MCA was used to detect and explore relationships between cognitive, clinical, physical, and lifestyle variables. Two PCA dimensions were identified (general cognition/executive function and memory), and two MCA dimensions were retained. Poorer cognitive performance was associated with older age, less school years, unhealthier lifestyle indicators, and presence of pathology. The first MCA dimension indicated the clustering of general/executive function and lifestyle indicators and education, while the second association was between memory and clinical parameters and age. The clustering analysis with object scores method was used to identify groups sharing similar characteristics. The weaker cognitive clusters in terms of memory and executive function comprised individuals with characteristics contributing to a higher MCA dimensional mean score (age, less education, and presence of indicators of unhealthier lifestyle habits and/or clinical pathologies). MCA provided a powerful tool to explore complex ageing data, covering multiple and diverse variables, showing if a relationship exists and how variables are related, and offering statistical results that can be seen both analytically and visually.

## 1. Introduction

Analysis of research data requires unique considerations depending on the type of data collected and/or on the main purpose of the research. For instance, while in some cases data is collected in ordinal mode, often it is also obtained in categorized groups. Or, as opposed to the traditional hypothesis testing designed to verify *a priori* hypotheses about relations between variables, exploratory data analysis is used to identify systematic relations between variables, when there are incomplete *a priori *expectations as to the nature of those relations. Falling in the latter category, the method correspondence analysis (CA), a (multivariate) descriptive data analytic technique, allows simplifying complex data and provides a detailed description of the data, yielding a simple, yet exhaustive analysis (a review of the development of the correspondence analysis methodology can be found in [1]). Specifically, multiple CA (MCA) allows for the analysis of categorical or categorized variables encompassing more than two categorical variables (whereas “simple” correspondence analysis pertains to the more “simple” dataset, a two-way contingency table) [2–7].

Summarily, MCA is part of a family of descriptive methods (e.g., clustering, factor analysis, and principal component analysis (PCA)) that reveal patterning in complex datasets. However, specifically, MCA is used to represent and model datasets as “clouds” of points in a multidimensional Euclidean space; this means that it is distinctive in describing the patterns geometrically by locating each variable/unit of analysis as a point in a low-dimensional space. The results are interpreted on the basis of the relative positions of the points and their distribution along the dimensions; as categories become more similar in distribution, the closer (distance between points) they are represented in space [2–6]. Although it is mainly used as an exploratory technique, it can be a particularly powerful one as it “uncovers” groupings of variable categories in the dimensional spaces, providing key insights on relationships between categories (i.e., multivariate treatment of the data through simultaneous consideration of multiple categorical variables), without needing to meet assumptions requirements such as those required in other techniques widely used to analyze categorical data (e.g., Chi-square analysis, Fischer's exact test, *G*-statistics, and ratio test) [8]. The use of MCA is, thus, particularly relevant in studies where a large amount of qualitative data is collected, often in pair with quantitative data, and where qualitative variables can become suboptimized in the data analysis. This is often the case in epidemiological and system studies where variables in the datasets may be quantitative or qualitative, temporal or nontemporal, and/or objective or subjective. As such, CA has been beneficial in areas ranging from the health and medicine to social sciences, archeology, ecology, software development, and market research (see reviews [7, 9, 10]).

In this context, among population-based studies, cognitive ageing studies can be particularly difficult to address. Foremost, data collection itself must consider and account for the multiple factors (variables) that might explain cognitive trajectories throughout ageing. Biologically, this is crucial because cognitive ageing results from a complex and adaptive interaction of endogenous and exogenous variables, ranging from physicostructural, clinical, genetic, and biochemical factors to psychological, social inclusion/continued intellectual stimulation, lifestyle, and sociodemographic indicators or measures [11–17]. In fact, altogether, these are at the basis of the inter- and intraindividual cognitive variability observed throughout ageing [18]. Subsequently, the large and diverse nature of the data renders analysis strategies decisions difficult. For example, while cognition is traditionally assessed via a battery of neurocognitive/psychological tests often on a continuous scale, other aspects, such as sociodemographic clinical variables, are often categorical or obtained in the form of questionnaires. Furthermore, even quantitative variables are often transformed into categorical ones (e.g., height and weight parameters to yield a BMI class). In this context, exploratory insights that maximize the use of all the qualitative information in the identification of categorical groupings of factors and reveal their relationship are needed if not crucial. In fact, illustrating its still surprisingly limited use albeit direct and valuable applicability in ageing studies, Sourial et al. [19] used MCA, encompassing data on several binary variables, from three separate studies, to examine the relationships among seven frailty domains in the elderly. The proof-of-concept study not only indicated that frailty is a multidimensional concept but also that MCA permits to efficiently gather separate large sets of data and/or to investigate for consistency between datasets, providing considerable insights in population studies.

Upon this, commonly used analysis strategies may be either unfeasible or when used alone only reveal that a relationship exists but not which response categories are related. For example, pairwise strategies are rendered impossible when dealing with a large number of categorical variables, and multivariate approaches (e.g., PCA) require the use of continuous variables. Furthermore, multivariate analysis results (despite their analysis power) do not allow exploring the individual response categories of the categorical variables. On the other end, MCA can account for these problems and preserve the categorical nature of the variables [2–6, 9]; the analysis is conducted at the level of the response categories themselves and not at the variable level. Furthermore, an important feature of CA is the graphical display of row and column points in biplots, which can help in detecting structural relationships among the variable categories and objects providing a visual map whose structuring can be interpreted (this duality is not present in other multivariate approaches to graphical data representation). Finally, CA has highly flexible data requirements, where the only strict data requirement is a rectangular data matrix with nonnegative entries. In fact, in a way, CA may be considered as a special case of PCA of the rows and columns of a table, especially applicable to a cross-tabulation; however, CA and PCA are used under different circumstances. Principal components analysis is used for tables consisting of continuous measurement, whereas correspondence analysis is applied to contingency tables (cross-tabulations). In CA the primary goal is to transform a table of numerical information into a graphical display, in which each row and each column are depicted as a point. The usual procedure for analyzing a cross-tabulation is to determine the probability of global association between rows and columns. The significance of association is tested by the Pearson chi-square test, but this test provides no information as to which are the significant individual associations between row-column pairs of the data matrix (i.e., it does not divulge how the association is constructed, nor does the statistic allow for an investigation of similar or different categories). Conversely, CA shows how the variables are related, not just that a relationship exists [2–6].

Based on the MCA technique, herein, we aim to obtain a global picture of the salient relationships among cognitive, clinical, physical, and lifestyle variables to explore their dimensional relationship to healthy ageing. The present work is based on the cognitive and clinical and sociodemographic assessment of older community-dwelling individuals in the Minho cohort in Northern Portugal [11, 20]. The study participants were representative of the Portuguese population in terms of age, gender, and educational status; on measures of sociodemographic characteristics the country ranks close to the OECD (Organisation for Economic Cooperation and Development; http://www.oecd.org/) average [21].

## 2. Material and Methods

### 2.1. Ethics Statement

The study was conducted in accordance with the Declaration of Helsinki (59th Amendment) and was approved by national and local ethics committees. The study goals and the neurocognitive, clinical, and lifestyle assessments were explained to potential participants. All volunteers provided a written informed consent. Further exclusion/inclusion selection criteria are described elsewhere [11, 12].

### 2.2. Sample Characteristics

Participants (*n* = 1051) were randomly selected from the Guimarães and Vizela local area health authority registries; however, the cohort was representative of the health registries (less than 2% difference) and of the general Portuguese population with respect to gender (females, *n* = 560 or 53.3%) and age (range: 50–97 years; M = 67.2, SD = 9.24; age categories: [50–60[, 25.4% (females, 52.8%); [60–70[, 31.2% (females, 53.7%); [70+[, 43.4% (females, 53.3%)). All participants were community-dwellers and the majority was in the medium socioeconomic stratum (61.6%, females 47.3%; Class III in the Graffar measure [22]) and retired (*n* = 763, females 51.8%). Literacy rate was 92.2% (able to read and write), and the median years of the schooling was 4; specifically, 34.7 (females 71.0%), 49.4 (females 47.4%), and 15.9% (females 32.9%) of the cohort attended school for [0–4[, 4, and [4+[ years. 

### 2.3. Neurocognitive Evaluation

Tests were selected to provide cognitive (general cognitive status and executive and memory functions) profiles, as previously reported [11, 12, 20]. The following measures were used: mini-mental state examination (MMSE) to assess global cognitive status [23]; digit span forward test (subtest of the Wechsler adult intelligence test WAIS III, 1997) to evaluate short-term verbal memory [24]; digit span backward test (subtest of the Wechsler adult intelligence test WAIS III, 1997) for verbal working memory [24]; the selective reminding test (SRT, parameters: consistent long-term retrieval (CLTR), long-term storage (LTS), delayed recall, and intrusions) to evaluate multiple trial verbal learning and memory [25]; Stroop color and word test (parameters: words, colors, and words/colors) to measure response inhibition/cognitive flexibility [26]; and the controlled oral word association test F-A-S (COWAT-FAS, parameters: admissible and nonadmissible) to assess verbal fluency [27]. A team of trained psychologists conducted the neurocognitive/psychological assessments.

### 2.4. Clinical, Physical, and Lifestyle Characteristics

General health aspects considered included clinical history of stroke (ischemic or hemorrhagic stroke or transient ischemic attack), cardiac pathology (this designation is here used to include coronary bypass, cardiac insufficiency, myocardial infarction, and/or coronary disease), diabetes (diabetes mellitus type I or II), dyslipidemia, and/or hypertension. Clinical measures are those self-reported by the participants in response to the standardized clinical interview. Physical measures included weight (Kg), height (m), and abdominal perimeter (cm). BMI (Kg/m^2^) was categorized as underweight, normal, overweight, and obese (resp., BMI: [0–18.5], [18.6–24.9], [25.0–29.9], and [30.0+[) [28]. For statistical procedures, the underweight and normal categories were combined due to the small sample size for underweight (*n* = 5). Metabolic complication risk was categorized none, increased, and substantially increased (resp., abdominal perimeter: females, [0–80.0], [80.1–88.0], and [88.1+[; males, [0–94.0], [94.1–102.0], and [102.1+[) [28]. Lifestyle, alcohol consumption (none, 50 or less, and more than 50 gr/day), physical activity status (none, less than 3, and over 3 times per week), and smoking habits (nonsmoker, former smoker, and smoker) were considered. A team of experienced clinicians performed a standardized clinical interview, including physical and lifestyle measures.

### 2.5. Analysis Methodology

Data analysis followed previously reported strategies [11, 12, 20] and was structured as follows.

1. Conversion of all neurocognitive test scores into *z* scores to express all variables in the same scale.
2. Exclusion of participants that met the previously established MMSE criteria for cognitive impairment (i.e., with a total score <17 if individual with ≤4 years of formal school education and/or ≥72 years of age or total score <23 if individual with more than 4 years of formal school education and/or ≤71 years of age).
3. Principal component analysis (PCA) for allocation of the neurocognitive multiple test parameters into single or composite cognitive dimensions.
4. Allocation of participants into cognitive categories according to quartile score for each of the identified PCA dimensions (below Q1 “poor”; middle 50% “normal”; above Q3 “good”).
5. Discretization of quantitative variables.
6. Multiple correspondence analysis (MCA) to explore the association between qualitative variables categories (cognitive, clinical, physical, and lifestyle).
7. Cluster analysis with object scores of each dimension to group subjects.
8. Crosstabulations with relevant variables in the MCA and cluster variable (proportion *z*-test).

The SPSS package v20 (IBM SPSS Statistics) was used to conduct all statistical analysis.

### 2.6. Principal Component Analysis (PCA)

PCA was used to reduce neurocognitive information through a linear function. All neurocognitive measures were considered in the analysis (extraction method: principal component analysis; rotation method: Varimax with Kaiser Normalization), and all individuals that met the established MMSE threshold and had no missing values in any of the considered neurocognitive measures (*n* = 684) were considered to identify the cognitive dimensions that grouped the neurocognitive variables. The parameters GDS, COWAT-FAS nonadmissible and SRT intrusions, and digit span forward were sequentially excluded from the analysis due to low component loadings (<0.400). The remaining parameters formed composites: “GENEXEC” (general cognition and executive function, Cronbach's alpha 0.793) composed of the parameters MMSE, Stroop (parameters: words, colors, and words/colors), FAS (parameter: admissible), and digits (parameter: backward); and “MEM” (memory function, Cronbach's alpha 0.890) composed of the SRT test variables (parameters: CLTR, LTS, and delayed recall). Next, we allowed and imputed values for the dimensions cases with only one missing value, yielding a total of *n* = 859 individuals with calculated scores in the identified cognitive dimensions (the sample was representative of the initial study population except regarding literacy rate, 99.4% able to read and write). Dimensions were calculated based on the weighted arithmetic mean of each cognitive test. The analysis followed and was in agreement with previously reported observations [11, 20].

### 2.7. Multiple Correspondence Analysis (MCA)

The object of correspondence analysis (CA) is to analyze categorical/categorized data that are transformed into cross tables and to demonstrate the results in a graphical manner. In CA, both relations between row and column variables and relations between different levels of each variable can be obtained [2–7]. Some considerations regarding CA are warranted particularly regarding the influence of cells and responses [1]. For instance, some reports have (i) explored the impact on the analysis by including and excluding/deleting categories [29, 30]; (ii) examined methods for identifying columns (attributes) that highlight row (incidence) differences [31]; and (iii) compared the theoretical similarities between CA and log-linear models [32]. Here, for MCA, from the initial total sample, *n* = 239 participants were excluded due to missing values in at least one of the considered clinical, general lifestyle, and/or physical variables, yielding, altogether, a total of *n* = 812 individuals (from the *n* = 859 from the cognitive analysis step) with no missing values which were included in all the remaining analysis. Since the missing values were assumed to be missing completely at random (MCAR) [33] and the range of the sample size was still above 250 after the exclusion of the missing data, this was considered the adequate strategy to follow, since there would be no prejudice to the adequacy, validity, or power of the present study [34, 35]. The sample remained representative of the initial study population for the measures considered.

Two solutions were explored using variable principal (VPrincipal) normalization method. The first solution included the maximum number of possible MCA dimensions (calculated from the difference between the sum of variables categories and the number of variables (39 minus 15, to yield 24 MCA dimensions)). The calculated total inertia was 1.6 (the maximum number of MCA dimensions (*n* = 24) divided by the number of variables (*n* = 15)). This step allowed exploring the number of dimensions to be included in the analysis and to obtain the reference value for total inertia (meaning that the contribution of each factor should now be calculated using the total inertia score as the denominator). The main use of inertia is as an indicator of the number of axes to retain for further analysis. To define the number of dimensions to retain, the following criteria/considerations were employed: (i) scree test [36]; (ii) eigenvalue (inclusion of MCA dimensions with inertia above 0.2 [3]); (iii) Cronbach's alpha score [3]; and (iv) although no defined number of dimensions is firmly established, some authors recommend two-dimensional pictures of data (which facilitates and allows for data interpretation) [37]. Based on these criteria, a second solution was explored with two MCA dimensions: the first accounting for 11.9% (0.190/1.6) of the variance and the second for 8.3% (0.132/1.6), yielding a total variance of 20.2% (0.323/1.6). Discrimination measures and a joint plot of category points were obtained. Category quantification plots constitute an alternative method of displaying discrimination of variables that can identify category relationships. The coordinates of each category on each dimension are displayed in order to determine which categories are similar for each variable. In the discrimination measures plot, the length and steepness of the lines indicate the discrimination measures of each variable for the two considered dimensions (another element for dimensions interpretation allowing to assess the relation system of the indicators and indicating its importance for each dimension). Specifically, in the MCA graphical representation, the squared distance of the *i*th row profile from the origin is

(1) $$\begin{array}{c} d_{I}^{2}\left(i,O\right)=\sum_{m=1}^{M*}f_{im}^{2} \end{array}$$

and is the Euclidean distance of the *i*th row profile coordinate from the origin [1], where, in the *M*-dimensional correspondence plot, the larger the distance of the *i*th row profile from the origin, the larger the weighted discrepancy between the profile of category *i* to the average profile of the column categories. As such, the further a point is from the origin, the greater the deviation from the expected under complete independence (a point near the origin indicates that the frequencies in row *i* of the contingency table fit the independence hypothesis). That is, the distance from an object to the origin is the reflection of the variation from the “average” pattern (the most frequent category for each variable). Objects with many characteristics corresponding to the average pattern lie near the origin, whereas objects with unique characteristics are located far from the origin (in this sense, the object scores plot is particularly useful for detecting outliers and typical groups of objects or revealing special patterns). Furthermore, by using confidence circles, it can be further graphically represented whether the position of a particular category contributes to the hypothesis of independence for the contingency table [38]. If the origin lies outside of the confidence circle, then the category can be considered to contribute to the dependency, whereas if the origin lies within the circle, it does not make such a contribution. The same conclusion can be made for the Euclidean distance of the column profile coordinates to the origin.

### 2.8. Cluster Analysis with Object Scores

Cluster analysis with object scores was used to classify subjects into groups (clustering variables: object scores of the MCA dimensions). Clusters are derived from the two MCA dimensions object scores. These values are based on the quantification of all qualitative variables (or treated as such) that define the individual profile. Since these are composite scores, the multidimensionality of the input (object scores) is preserved when performing cluster analysis. The “method of reciprocal averaging,” that marks the MCA approach of the Leiden University in IBM SPSS software, is used to transform objects and variables categories. This method relates the quantifications between the variable categories and the object scores. The quantification of a certain category is the average of all the respective objects, and each object score is proportional to the average of all categories that the object is associated with. The quantification of the *p* categories of the *m* variables is calculated based on the formula *Y* = *D* ^−1^ *G*′*X*, where *Y* is the categories quantification matrix; *D* is frequency of the *p* category; *G* is the binary matrix; and *X* is the object score matrix. Cluster analysis defines homogeneous subjects profiles based on the MCA dimensions assuming that they have substantive coherence.

Four separate clustering solutions, comprised of 2–5 clusters, were tested. Three criteria were considered to choose the best cluster solution: (i) the solution explained ≥5% of the dependent variables compared to the previous applied solution; (ii) the variance was greater than that of the previous solution; and (iii) the individuals were evenly distributed among the clusters (for each cluster solution). ANOVAs were performed on each cluster solution, using the MCA dimensions as dependent variables and cluster membership as a factor variable (independent variable). General effect size *η* ^2^ was derived by dividing the sum of all between-groups sum of squares by the sum of the total sum of groups; for the 2-, 3-, 4-, and 5-cluster solutions, *η* ^2^ was 0.33, 0.60, 0.74, and 0.77, respectively. While an increase in cluster solutions corresponded to an increase in variance, the five-cluster solution only explained 3.6% more than the four-cluster solution, compared with the four-cluster solution explaining 13.7% more than the three cluster solution. In the four-cluster solution, group membership varied between 20.8% (*n* = 169) and 27.6% (*n* = 224). The 4-membership clustering solution was considered to provide the best cluster solution.

## 3. Results

### 3.1. Multiple Correspondence Analysis

**Figure 1:**
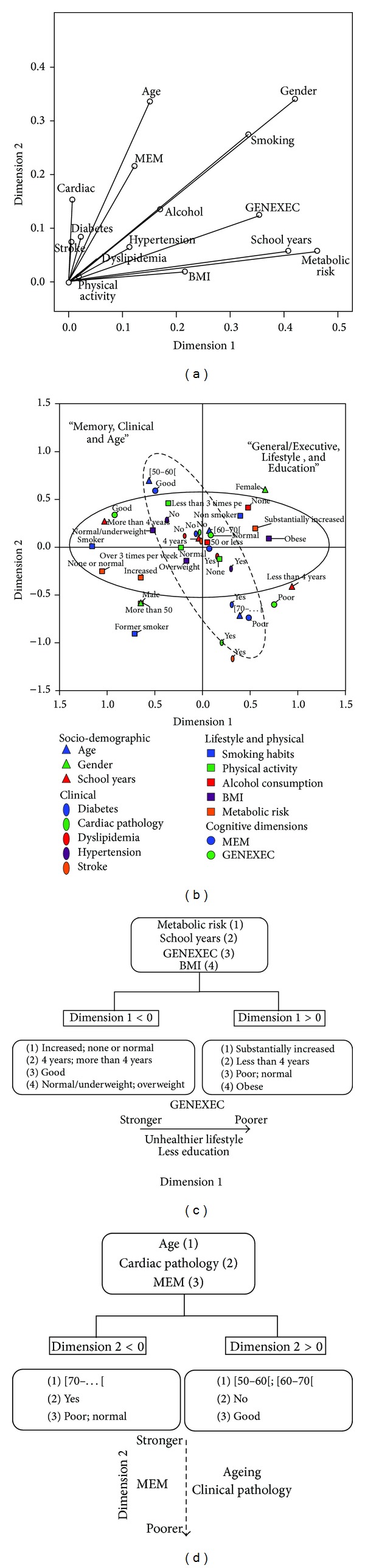
MCA dimensions. (a) MCA dimensions discrimination measures. (b) Joint category plot of the explored variable categories. (c) Positive and negative centroid coordinates for dimension 1. (d) Positive and negative centroid coordinates for dimension 2.

Clinical, general lifestyle, physical, and cognitive characterization of the MCA study sample (*n* = 812) is presented in Table 1. From the MCA analysis, a two-dimension MCA solution was considered the most adequate. The first and second dimensions presented are, respectively, eigenvalue, 2.857 and 1.984; inertia, 0.190 and 0.132; and Cronbach's alpha, 0.696 (95% CI 0.665, 0.726) and 0.531 (95% CI 0.483, 0.577). Although the generally accepted lower limit for Cronbach's alpha is 0.70, a smaller value is acceptable in exploratory research [2] where a small alpha score can be due to a reduced number of questions, poor interrelatedness between items, or heterogeneous constructs. Here, we are dealing with heterogeneous constructs to capture a two-dimensional picture of the data, and the methodological procedure was conducted assuming for this limitation. Discrimination measures (Table 2 and Figure 1(a)) and a joint plot of category points were obtained (Figure 1(b)). There were no clear differentiating values allocated to each of the obtained dimensions (Table 2); all discrimination measures were below 0.5 with a maximum value of 0.462 (metabolic risk) for the first dimension and 0.350 (gender) for the second dimension. Gender also contributed to the eigenvalue of the first dimension (value 0.419). The most discriminant variables for dimension 1 hierarchically were metabolic risk, school years, general cognition/executive function, and BMI; regarding dimension 2, the most discriminant variables were age, cardiac pathology, and memory (Table 1 and Figures 1(a)–1(d)). The variables gender, smoking, and alcohol presented relevant and similar discrimination measures in both dimensions. From the results and their graphical visualization, dimension 1 was termed “General/Executive, Lifestyle, and Education” and the second dimension “Memory, Clinical, and Age.” 

In dimension 1, gender correlated (transformed variables) significantly with smoking (*r* = 0.583, *P* < 0.001), alcohol (*r* = 0.459, *P* < 0.001), and metabolic risk (*r* = 0.514, *P* < 0.001); age correlated with school years (*r* = 0.334, *P* < 0.001), GENEXEC (*r* = 0.304, *P* < 0.001), and MEM (*r* = 0.316, *P* < 0.001); school years with GENEXEC (*r* = 0.452, *P* < 0.001); BMI correlated with metabolic risk (*r* = 0.529, *P* < 0.001); and GENEXEC correlated with MEM (*r* = 0.380, *P* < 0.001). Similar correlations were found for dimension 2, except for BMI with metabolic risk where no correlation was found. Only correlations above 0.30 were considered to have meaningful practical significance.

### 3.2. Cluster Analysis

**Figure 2:**
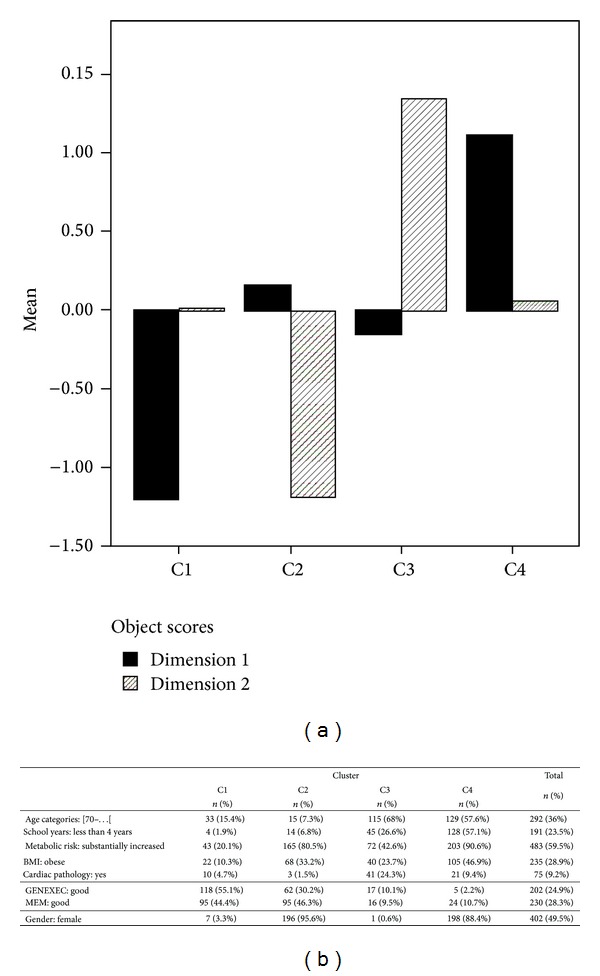
Cluster analysis with object scores. (a) Clusters (clusters 1 to 4, C1 to C4) identified for the MCA dimensions “General/Executive, Lifestyle, and Education” (dimension 1) and “Memory, Clinical, and Age” (dimension 2). (b) Crosstabulations with relevant variables in the MCA (and gender) and cluster variable.

The clustering analysis with object scores method was used to identify groups sharing similar characteristics within each of the identified dimensions (“General/Executive, Lifestyle, and Education” and “Memory, Clinical, and Age”). Specifically, analysis revealed 4 distinct clusters (cluster 1 to 4, C1 to C4) for each dimension (Figure 2). For GENEXEC, the different clusters showed a progressive decrease in performance (C1 > C2 > C3 > C4, all significantly different from each other; *z*-test for proportions comparisons adjusted with Bonferroni method, *P* < 0.05), while for MEM, the clusters C1 and C2 were comparable and the clusters C3 and C4 as well (C1 = C2 > C3 = C4, no significant difference between C1and C2 and between C3 and C4, but significant difference between C1/C2 and C3/C4 was found, *P* < 0.05). The relevant variables in the MCA dimensions were next cross-tabulated with cluster variable. Regarding age categories, C1 and C2 were significantly different from C3 and C4, specifically the proportion of older participants “[70+[” was significantly higher in the latter two clusters (C4 = C3 > C2 = C1, *P* < 0.05). A similar pattern was present regarding school education; specifically, for the “less than 4 school years” category C4 > C3 > C2 = C1 (*P* < 0.05). For “metabolic risk significantly increased,” all clusters significantly differed from each other (C4 > C2 > C3 > C1, *P* < 0.05), with a similar pattern noted for “BMI obese” (C4 > C2 = C3 > C1, *P* < 0.05). Finally, for presence of “cardiac pathology,” C3 > C4, C2, and C1, with C4 > C2 and = C1 and C2 = C1. Although not a discriminant variable in MCA, interestingly for gender the proportion of females was significantly higher in C2 and C4 compared to C1 and C3 (*P* < 0.05).

## 4. Discussion

Correspondence analysis is a technique that represents graphically the row and column categories and allows for a comparison of their “correspondences” (associations) at a category level. The development of CA has not been exclusively confined to statisticians; its diversity of development and application range has allowed for its application in, for example, the fields of health, social sciences and archaeology [1]. As such, altogether, CA makes a very relevant method of data analysis when an exploratory or even more in-depth analysis of categorical data is required, making it a particularly useful technique as it (i) is versatile, in part because no underlying distributional assumptions are required, thus accommodating any type of categorical variable whether binary, ordinal, or nominal; (ii) gives a graphical output (often two-dimensional) for representing the associations between the variables in a low-dimensional space, thus providing key exploratory insights on the relationships between the collected data; and (iii) can be a complement or used in pair with other methods such as multidimensional scaling, biplots, and PCA (strategies followed in this report) [2–6]. 

Herein, we used a combined approach of PCA and MCA to a cross-sectional analysis in order to, upon identifying main cognitive dimensions, explore relationships between cognitive, lifestyle, physical, and clinical variables among community-dwelling older individuals. The combination of the two methodologies is here favorable, with neither method used in detriment of the other but rather complementarily. First, PCA allowed grouping neurocognitive test variables (measures of cognition, each retaining a calculated weight in the final dimension) into cognitive dimensions, subsequently permitting the establishment of the “cognitive” score/class of each individual in regard to the entire cohort. Following previous findings [11, 20], two main cognitive dimensions were identified that represented general cognition and executive function (GENEXEC) and memory (MEM), each of which forming composites of multiple neurocognitive test variables. Thereafter, cognitive performance (categorized as “poor,” “normal,” and “good”) could be explored in relation to the mixture of binary, categorical, discrete, or continuous variables (these suitably categorized) that comprised the sociodemographic, clinical, lifestyle, and physical aspects. Evidence of these relationships is necessary to elucidate whether particular characteristics belong to the construct of stronger versus poorer cognitive performance, with foreseeable value in our ageing society. In fact, albeit today's older adults being generally considered healthier and, likely consequently, more independent and active—with a significant positive implication in their continued contribution to society (particularly if with an accompanying proper allocation/restructuring of resources, policies, and interventions [39]), an increase in concurrent medical conditions can have detrimental consequences, causing excess (co)morbidity, disability, and decline in functional performance [40]. Furthermore, even if considering that gender, educational, and clinical aspects may remain “fixed” parameters (e.g., an individual either has or not a cardiac pathology), their association/combinatory effect with other more “modifiable” lifestyle parameters (e.g., physical activity) is of relevant interest. 

From data analysis, and its graphical representation, two MCA dimensions—termed “General/Executive, Lifestyle, and Education” and “Memory, Clinical, and Age”—were identified. For the first, a more unhealthy lifestyle (as indicated by higher BMI and metabolic risk, among other measures) and lower education level were associated with poorer general cognition and executive function, while clinical aspects and ageing itself appeared to cluster with memory performance in a second dimension. Cluster analysis further added to the findings; with an increase in the overall dimension (object) score (meaning the contribution of age, pathologies, and indicators of more unhealthy lifestyle factors), the overall cognitive performance decreased. Particularly, when clusters were analyzed for the relevant measures, the weaker clusters in terms of cognition (C3 and C4), across memory and executive function domains, were those that comprised a combination of older participants, with lower school education, obese, with substantially increased metabolic risk, and with presence of cardiac pathology. Interestingly, while executive function appeared more particularly susceptible to indicators of an unhealthier lifestyle measures (which is particularly interesting in the case of C1 and C2 clusters, where, with no significant differences in age and school years, the GENEXEC performance is significantly different, together with indicators of lifestyle), memory seemed equally sensitive to these and to the presence of clinical pathology (especially, cardiovascular pathology). While pathologies such as diabetes, metabolic syndrome (and its individual components), and vascular-related pathologies have been associated with cognitive decline, deficits, and/or impairment, the maintenance of a “healthy” lifestyle and introduction of beneficial interventional measures have been associated with cognitive improvements, including the overall pathology and isolated disease components [41–46]. Specifically, risk factors for cerebrovascular and cardiovascular pathologies or disease are thought to reduce blood flow to the frontal and subcortical brain regions and therefore impact (negatively) cognitive function [47]. Additionally, the lifestyle indicators, BMI, and metabolic risk have been associated with memory performance [48, 49]. However, it is the interdependency/relation between the variables, possibly explaining unique trajectories of cognitive ageing, which actually enforces the need for encompassing exploratory studies and appropriate methodology. For instance, unhealthy behavior can act as a risk factor for chronic disease itself with impact on negative mood that, in turn, can trigger further unhealthy habits and consequently worsening of chronic conditions [50].

These observations are in line with a recently published longitudinal study indicating that the combined effect of multiple risk factors may be of greater concern than individual triggers on cognitive decline in older adults [51]. Specifically, it was shown that smoking had the most consistent longitudinal impact linked with lower cognitive performance on multiple cognitive outcomes (including memory and executive function). Here, interestingly, the variable smoking had relevant and similar discrimination measures in both dimensions, followed by alcohol. Regarding alcohol, published data relating its intake with cognition among older adults is mixed. On one hand, studies comparing drinkers and nondrinkers report lower cognitive functioning among nondrinkers and heavier drinkers compared to “average” (7–14 drinks per week) drinkers (e.g., [52]); however, even among longitudinal studies reporting statistically significant associations between better cognition and moderate alcohol consumption, the magnitudes of the associations can be small. Another limitation is that domain-specific measures of cognition are not often employed (as discussed, [53]). Still, recent work, which addresses these drawbacks, indicates that moderate alcohol intake through midlife and into later life confers the best cognitive outcomes in old age, as defined by word-finding ability. The relationships were independent of age, smoking status, hypertension, and gender. The authors also indicate that heavy drinkers had the lowest phonemic fluency scores, which is consistent with other tests of executive function and may precede declines in memory [53]. Finally, it was interesting that here gender itself was not a relevant measure in MCA and neither was it the variable that discriminated between clusters of performance (cluster analysis). In fact, the impact of gender on cognitive ageing is not clear in the literature. Studies that appear contradictory may simply just indicate that age classes may particularly matter when considering gender (e.g., regarding neuroendocrine aspects or menopause [54]), and/or that gender may have an indirect effect. For instance, if in the past females had a more secondary role in terms of participation in society and/or access to education, this might be manifested in poorer cognitive performance compared with the male peers, including the older years.

Following other studies across a breadth of epidemiological research that either also used MCA as the primary analysis tool or as a basis to building statistical models (e.g., [55–61]), here the strategy was meaningful, yielding results in agreement with the literature, revealing that MCA is a relevant methodological approach for a valuable first insight into medium-size datasets consisting of multiple domains (e.g., cognitive, clinical, and sociodemographic). Nonetheless, a few considerations are warranted. Although one of the advantages of the MCA technique is that qualitative information is transformed into quantitative information to be used in further analysis, when quantitative variables are transformed into qualitative ones some of its properties may be lost as well as the measurement precision. However, unlike most complex statistical methods, for the MCA procedure there are not any preconditions (such as multivariate normality and linearity). This technique allows the analysis of the relations between variables and between different categories/levels of each variable, offering at the same time, in comparison to other methods, statistical results that can be seen both analytically and visually. Furthermore, since it is based on the variable categories (distance), the directionality of the relationship is not applicable. Nonetheless, CA does remain an exploratory tool for the analysis of association(s) between categorical variables. Finally, here, the analysis is cross-sectional; however, CA can equally be used in longitudinal data. When there is one categorical variable measured at two-time points, a transition matrix can be constructed; in this case, the aim of a correspondence analysis of a transition matrix is to get an insight into the transitions from time 1 to time 2. Given that different questions about these transitions exist, these lead to different forms of CA (the same reasoning applies to CA of more than two time points). We refer to the Van der Heijden 2005 [62] article for more details on the applicability and considerations of CA in longitudinal studies.

The methodology should now be replicated across European cohorts to explore the findings in other study populations. Findings are expected to have direct implications in the clinic, identifying groups at risk for cognitive decline and decrease in functionality.

## Figures and Tables

**Table 1:** Clinical, general lifestyle, physical, and cognitive characterization.

	Count	Column *n* (%)
*Sociodemographic characteristics *		
Gender		
Male	410	50.5%
Female	402	49.5%
Age		
[50–60[	239	29.4%
[60–70[	281	34.6%
[70–...[	292	36.0%
School years		
Less than 4 years	191	23.5%
4 years	465	57.3%
More than 4 years	156	19.2%
*Clinical characteristics *		
Stroke		
Yes	42	5.2%
No	770	94.8%
Cardiac pathology		
Yes	75	9.2%
No	737	90.8%
Diabetes		
Yes	153	18.8%
No	659	81.2%
Dyslipidemia		
Yes	449	55.3%
No	363	44.7%
Hypertension		
Yes	452	55.7%
No	360	44.3%
*Lifestyle and physical characteristics *		
Smoking habits		
Former smoker	200	24.6%
Smoker	63	7.8%
Nonsmoker	549	67.6%
Alcohol consumption		
50 or less gr/day	379	46.7%
More than 50 gr/day	200	24.6%
None	233	28.7%
Physical activity		
Less than 3 times per week	134	16.5%
Over 3 times per week	178	21.9%
None	500	61.6%
BMI		
Normal/underweight	188	23.2%
Overweight	389	47.9%
Obese	235	28.9%
Metabolic risk		
Increased	199	24.5%
Substantially increased	483	59.5%
None	130	16.0%
*Cognitive dimensions *		
GENEXEC		
Poor	201	24.8%
Normal	409	50.4%
Good	202	24.9%
MEM		
Poor	176	21.7%
Normal	406	50.0%
Good	230	28.3%

**Table 2:** MCA dimensions discrimination measures.

	MCA dimension		Mean
	1	2	
Gender	0.419	0.350	0.384
Age	0.150	0.332	0.241
School years	0.409	0.063	0.236
Stroke	0.005	0.073	0.039
Cardiac	0.006	0.139	0.073
Diabetes	0.022	0.083	0.053
Dyslipidemia	0.029	0.011	0.020
Hypertension	0.110	0.061	0.086
Smoking habits	0.333	0.272	0.302
Alcohol consumption	0.169	0.136	0.152
Physical activity	0.052	0.048	0.050
BMI	0.216	0.017	0.117
Metabolic risk	0.462	0.053	0.258
Cognitive dimension GENEXEC	0.352	0.125	0.239
Cognitive dimension MEM	0.122	0.221	0.171

Active total	2.857	1.984	2.421
% of variance	19.045	13.229	16.137
